# Novel intrathoracic irrigation using ultrafine ozone bubbles in a rat empyema model

**DOI:** 10.1038/s41598-023-43787-3

**Published:** 2023-10-10

**Authors:** Masaki Ikeda, Yojiro Yutaka, Toyofumi F. Chen-Yoshikawa, Michio Tanaka, Masaki Yamamoto, Satona Tanaka, Yoshito Yamada, Akihiro Ohsumi, Daisuke Nakajima, Masatsugu Hamaji, Akihiko Yoshizawa, Eishi Kusaka, Miki Nagao, Hiroshi Date

**Affiliations:** 1 Department of Thoracic Surgery, Graduate School of Medicine, Kyoto University, 54 Shogoin Kawahara‑cho, Sakyo‑ku, Kyoto, 606‑8507 Japan; 2 Department of Thoracic Surgery, Graduate School of Medicine, Nagoya University, Nagoya, Japan; 3 Department of Clinical Laboratory Medicine, Graduate School of Medicine, Kyoto University, Kyoto, Japan; 4 Department of Diagnostic Pathology, Kyoto University Hospital, Kyoto, Japan; 5 Department of Energy Science and Technology, Graduate School of Energy Science, Kyoto University, Kyoto, Japan

**Keywords:** Microbiology, Diseases, Materials science

## Abstract

Dissolved ozone is generally used for sanitization, but it has not been used for thoracic cavity sanitization because of its short half-life (< 20 min) and possible toxicity. We developed a novel solution containing ultrafine ozone bubbles (ozone-UFB) with a fivefold longer half-life than non-UFB ozone. Using an in vitro model, *Staphylococcus aureus* colonies were counted after exposure to ozone-UFB or non-UFB ozone at the same ozone concentration (0.4 mg/L). The colony count was significantly lower in the ozone-UFB group than in the non-UFB ozone group (*p* = 0.034). The effect of repeated pleural irrigation using ozone-UFB and saline was compared in a rat empyema model of *S. aureus* infection. The bacterial count in the pleural effusion was decreased by at least fivefold following intrathoracic lavage with ozone-UFB (3 mg/L). To examine the safety of ozone-UFB for intrathoracic use, ozone-UFB with a higher ozone concentration (10 mg/L) was injected into the thoracic cavities of normal rats. The treatment did not result in any specific pleural damage or elevated serum interleukin-6 concentrations. The findings highlighted the efficacy and safety of ozone-UFB for intrathoracic sanitization, but further studies are needed to determine the optimal therapeutic ozone concentration with appropriate safety margins.

## Introduction

Empyema is generally defined as a pathological condition in which pus accumulates in the thoracic cavity. Empyema is associated with pneumonia, chest surgery, trauma, and esophageal perforation. In the USA, 1 × 10^6^ people are hospitalized for pneumonia each year. Of these, 20%–40% have pleural effusion and 5%–10% of those with pleural effusion develop empyema^1^. In Japan, pleural effusion occurs in 30%–40% of people with bacterial pneumonia and 0.5%–2% of people with pleural effusion develop empyema^2^.

Local treatment, such as drainage, is useful because antibacterial agents that are administered usually reach low concentrations in the thoracic cavity. However, the effects of intrathoracic irrigation are not well investigated. One reason for this is that normal saline is the only safe liquid available for intrathoracic irrigation. Reports of human clinical studies show that povidone-iodine is effective for empyema and pleural infection^3,4^. Moreover, pleurodesis using povidone-iodine against air leaks following lung surgery, malignant pleural effusion, and chylothorax has been successful, with the occurrence of only non-serious adverse events such as chest pain^5–7^. However, in Japan, it is currently not approved for use in body cavities because of concerns about tissue damage and residues^8^. Additionally, chlorhexidine is only approved as a skin sanitizer in Japan^9^.

Here, we focused on using ozone dissolved in water to clean the thoracic cavity. Ozone dissolved in water has well-known bactericidal effects, and the effects on various microorganisms have been reported^10,11^. Ozone dissolved in water has been safely used to sterilize the surfaces of foods such as vegetables and fruits^12^. However, ozone dissolved in water has a high deactivation rate at 20 °C and has a half-life of 20–30 min, so an ozone solution needs to be prepared immediately before clinical use^10,13,14^. Ozone dissolved in water is readily deactivated by organic matter^10^. A technique for preparing a liquid containing bubbles with diameters of 100–200 nm, called ultrafine bubbles (UFBs), has recently been developed. Ozone UFBs can remain dissolved and stable for longer than non-UFB ozone^13^. Solutions containing ozone-UFBs have been used in the medical field, and there have been several publications about the efficacy and safety of using ozone-UFBs to treat periodontal disease^15,16^. However, no studies of the efficacy and safety of using ozone-UFBs within the body cavity have been performed. Furthermore, the effects of solutions containing ozone-UFB in the presence of organic matter are unclear.

We assessed the bactericidal effects of a solution containing ozone-UFBs in the presence of organic matter in vitro, the bactericidal effects of a solution containing ozone-UFBs in a rat empyema model, and the effects of a solution containing ozone-UFBs injected into the thoracic cavities of rats on tissue damage.

## Materials and methods

### Preparing a solution containing ozone-UFBs and determining the ozone concentration

An ozone-UFB solution was prepared using a two-step procedure. First, oxygen was separated from air and ozone was created from the oxygen using a silent electric discharge system in a PZH-12N ozone production device (KOFLOC, Kyoto, Japan). Second, ozone-UFBs were created in a normal saline solution (NSS; Otsuka Pharmaceutical, Tokyo, Japan) by dissolving the ozone by applying power and causing cavitations to form using a UFB creation device (LIVING ENERGIES & Co., Shizuoka, Japan). There were ~ 3 × 10^8^ UFB particles per milliliter of solution, and the UFB diameters were 100–200 nm. The number of UFBs remained sufficient for the tests for > 3 days. The number of UFBs was determined using a NanoSight system (Malvern Panalytical, Malvern, UK). The UFB size distributions are shown in Supplemental Fig. 1. The dissolved ozone concentration in the ozone-UFB NSS decreased more slowly than the dissolved ozone concentration in NSS containing non-UFB ozone at room temperature (22 °C), as shown in Supplemental Fig. 2. Supersaturated ozone rapidly volatilized in the first 10 min after the ozone-UFB NSS was produced, but the ozone concentration then decreased slowly and remained ~ 1 mg/L for > 1 h. In contrast, almost no ozone was detected in the NSS containing non-UFB ozone after another 10 min.

### Time to achieve disinfection

#### Microorganisms

Several microorganisms cause empyema^17^. *Staphylococcus aureus ATCC 29213* (*S. aureus*^*ATCC*^) and *Pseudomonas aeruginosa ATCC 27853* (*P. aeruginosa*^*ATCC*^) used in this study were obtained from the American Type Culture Collection (ATCC). Methicillin-resistant *S. aureus* (MRSA) was obtained from a clinical sample from Kyoto University Hospital (MRSA*-Kyoto*). *Peptostreptococcus anaerobius GTC 0201* (*P. anaerobius*^*GTC*^) was obtained from Gifu University Center for Conservation of Microbial Genetic Resourcethrough the National BioResource Project (Pathogenic Bacteria) run by MEXT/AMED (Japan). *S. aureus*^*ATCC*^, *P. aeruginosa*^*ATCC*^, and MRSA*-Kyoto* were grown on trypticase soy agar (BD, Franklin Lakes, NJ, USA) at 35 °C for 1 day. *P. anaerobius*^*GTC*^ was grown anaerobically on GAM agar (Nissui Pharmaceutical, Tokyo, Japan) at 36 °C for 3 days.

#### Sample preparation

A 0.1 mL aliquot of culture medium containing ~ 1 × 10^7^ colony forming units (CFUs)/mL of test bacteria was added to 0.9 mL of ozone-UFB NSS at one of several concentrations (prepared from a solution with an initial ozone concentration of 0.4 mg/L) at 25 °C. The bactericidal effect of the ozone-UFB NSS was compared with the bactericidal effect of 0.5% chlorhexidine gluconate (CHX) (0.5% Hexizac Water W; Yoshida Pharmaceutical, Saitama, Japan).

The mixture was then incubated at 25 °C for 1 min. In tests to compare the bactericidal effects of ozone-UFB NSS and CHX, the bacterial suspensions were incubated for either 1 or 10 min because the duration of the bactericidal effect of each solution was different. CHX did not become deactivated in the mixed solution but ozone-UFB NSS rapidly became deactivated. The bactericidal effects of ozone-UFB NSS on *P. anaerobius*^*GTC*^ and air-UFB NSS were also compared.

#### Counting bacterial colonies after exposure to a test solution

After incubation, a 0.1 mL aliquot of a mixture was aseptically transferred to a Petri dish and mixed with ~ 20 mL of trypticase soy agar medium, then the mixture was incubated at 35 °C for 1 day (for *S. aureus*^*ATCC*^, *P. aeruginosa*^*ATCC*^, and MRSA*-Kyoto*) or mixed with 20 mL of GAM agar medium and incubated at 36 °C for 3 days under anaerobic conditions (for *P. anaerobius*^*GTC*^). In the tests performed to compare the ozone-UFB and CHX solutions, a 0.1 mL aliquot of a mixture was aseptically transferred to a Petri dish and mixed with 20 mL of tryptic soy agar containing polysorbate 80 and lecithin (Nissui Pharmaceutical) to deactivate the test solution, then the mixture was incubated at 35 °C for 2 days. The colonies that formed were then counted. The minimum bactericidal concentration (MBC) was calculated. The MBCs for *S. aureus*^*ATCC*^ and *P. aeruginosa*^*ATCC*^ for non-UFB ozone solutions and ozone-UFB solutions were compared.

#### Bactericidal effects of ozone-UFB solutions for solutions containing bacteria and albumin

Dissolved ozone is readily deactivated by organic matter, so we assessed the bactericidal effects of ozone-UFB solutions in solutions containing *S. aureus*^*ATCC*^ and albumin in in vitro tests imitating the intrathoracic environment of empyema.

##### Bactericidal effects of ozone-UFB solutions at different albumin concentrations

A 50 µL aliquot of culture medium containing ~ 10^7^ CFUs/mL of *S. aureus*^*ATCC*^ was mixed with NSS containing bovine albumin (Serologicals Corporation, Norcross, GA, USA) at a concentration of 10%, 5%, 1%, or 0.1%. A 0.1 mL aliquot of the mixture was then added to 0.9 mL of ozone-UFB solution (ozone concentration 0.5 mg/L), then the mixture was incubated at 25 °C for 10 min. A 0.1 mL aliquot of the mixture was then aseptically transferred to a Petri dish and mixed with 20 mL of trypticase soy agar medium, then the mixture was incubated at 35 °C for 1 day and the colonies were counted.

##### Bactericidal effects of ozone-UFB solutions added several times to a solution containing *S. aureus* and albumin

A 50 µL aliquot of culture medium containing ~ 10^8^ CFUs/mL of *S. aureus*^*ATCC*^ was mixed with NSS containing bovine albumin at a concentration of 10%. A 0.1 mL aliquot of the mixture was then added to 0.9 mL of ozone-UFB solution (ozone concentration 0.7–1.0 mg/L). A 0.1 mL aliquot of the mixture was then transferred to a tube containing 0.9 mL of ozone-UFB solution, and the same dilution was repeated two more times. For each mixture, a 0.1 mL aliquot was aseptically transferred to a Petri dish and mixed with 20 mL of trypticase soy agar medium, then the mixture was incubated at 35 °C for 1 day. The colonies were then counted.

### Rat empyema model

#### Preparation

A controlled experiment was performed at the Institute of Laboratory Animals, Graduate School of Medicine, Kyoto University, Kyoto, Japan. The study protocol and all procedures were approved by the Kyoto University Ethics Committee (approval number Med Kyo 21306). The animals received humane care in compliance with the Principles of Laboratory Animal Care outlined in Federal Resolution 04/97. This study was carried out in compliance with the ARRIVE guidelines. Male Lewis rats each weighing 280–330 g were randomly selected. The rat supplier was Japan SLC (Hamamatsu, Japan). The effects of intrathoracic lavage using ozone-UFBs were compared with the effects of lavage using NSS using a rat empyema model using *S. aureus*^*ATCC*^ (one of the most common causes of empyema). The rat empyema model was created by performing intrathoracic injection of 1.0 mL of NSS containing bacteria (~ 10^8^ CFUs of *S. aureus*^*ATCC*^). The bacteria solution was injected into the right thoracic cavity through the diaphragm by performing a small laparotomy to avoid postoperative adhesion. All procedures were performed using aseptic techniques.

#### Treatment and assessment

Two days after *S. aureus*^*ATCC*^ had been injected, rats with sufficient pleural purulent effusion (≥ 0.5 mL) in which *S. aureus*^*ATCC*^ was detected after culturing the liquid were defined as having had empyema. Because further survival was not expected in this empyema model, all of the rats were sacrificed by causing them to inhale a lethal dose of isoflurane. The rats were placed in the lateral decubitus position. Skin disinfection was performed with 70% ethanol followed by 10% povidone-iodine. The following procedures were conducted in an aseptic manner using sterilized material. Right thoracotomy with resection of several ribs was performed to allow the entire thoracic space to be inspected. Pleural effusion occurred in both sides of the thoracic space, so the mediastinal pleura was removed to allow all of the pleural effusion to be collected from both sides.

After the effusion had been collected, lavage was performed twice, each time using 3 mL of urokinase (Mochida Pharmaceutical, Tokyo, Japan) diluted with NSS to give 6000 units/mL for 10 min, to remove suspended organic matter. The thoracic cavity was then filled for 2 min with ozone-UFB NSS or NSS. This was repeated 15 times. Ozone-UFB NSS with an ozone concentration of 3–4 mg/L was used for the lavage procedure. Once lavage had been performed 15 times, the thoracic cavity (of each rat in both groups) was filled for 2 min with NSS. The liquids from the first, fifth, 10th, and last NSS lavages were cultured. The pleural effusion and urokinase lavage liquids were each diluted by a factor of 100 and the immersion liquids were each diluted by a factor of 10 with NSS. A 0.1 mL aliquot of a sample was aseptically transferred to a Petri dish and mixed with 20 mL of trypticase soy agar medium, then the mixture was incubated at 35 °C for 1 day. The colonies were then counted. The decreases in the numbers of *S. aureus*^*ATCC*^ in the first, fifth, 10th, and last NSS immersion liquids were determined and compared with the total bacterial counts for the initial pleural effusion and urokinase lavage liquids.

### In vivo toxicity study

Laparotomies were performed in the same manner as the empyema model using male Lewis rats weighing 280–330 g. A 1.0 mL aliquot of NSS containing ozone-UFBs at ozone concentrations of 9–10 mg/L or a 1.0 mL aliquot of NSS (the control) was injected intrathoracically through the diaphragm. The incision was closed without collecting the injected liquid, and the toxicity was evaluated 24 h later.

The rats were sacrificed 24 h after the liquid was injected, and a blood sample was obtained from the inferior vena cava of each rat. The interleukin-6 (IL-6) concentrations in the serum samples were determined and used to indicate inflammation. The IL-6 concentrations were determined by performing enzyme-linked immune sorbent assays using LEGEND MAX™ Rat IL-6 ELISA kits (Biolegend, San Diego, CA, USA). The lower right lung lobe, heart, liver, and right kidney were resected for histopathological examination to allow inflammatory changes to be evaluated. The tissue samples were fixed in 10% formalin and stained with hematoxylin–eosin. Each rat was weighed before the intervention and 24 h later.

### Statistical analysis

Each result is presented as the median and interquartile range unless otherwise stated.

The results for pairs of groups were compared by performing Fisher’s exact tests for categorical data and Mann–Whitney *U* tests for nonparametric variables. Wilcoxon signed rank tests were used to compare the MBCs for *S. aureus*^*ATCC*^ and *P. aeruginosa*^*ATCC*^ for non-UFB ozone and ozone-UFB solutions. *P* < 0.05 was considered to indicate a statistically significant result. Statistical analyses were performed using EZR statistical software (Saitama Medical Center, Jichi Medical University, Saitama, Japan).

## Results

### Time to achieve disinfection

#### Enumeration of bacterial colonies following exposure to each solution

**Figure 1:**
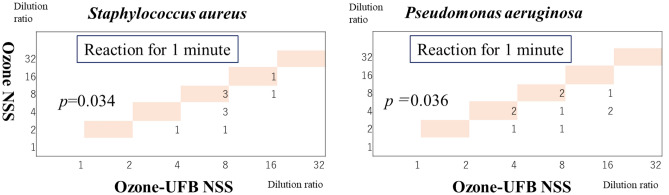
Minimum bactericidal concentrations for *Staphylococcus aureus* (ATCC 29213) and *Pseudomonas aeruginosa* (ATCC 27853) for a normal saline solution (NSS) containing ozone and ultrafine bubbles of ozone (ozone-UFB) in NSS relative to the minimum bactericidal concentration distribution tables.

The median MBCs for *S. aureus*^*ATCC*^ and *P. aeruginosa*^*ATCC*^ for ozone-UFB NSS in 1 min were both 1/8 (range 1/4 to 1/16). The MBCs for *S. aureus*^*ATCC*^ and *P. aeruginosa*^*ATCC*^ for non-UFB ozone and ozone-UFB in NSS and the relative MBC distribution tables are shown in Fig. 1. The MBCs for *S. aureus*^*ATCC*^ and *P. aeruginosa*^*ATCC*^ were lower for ozone-UFB NSS than non-UFB ozone NSS (*p* = 0.034 for *S. aureus*^*ATCC*^ and *p* = 0.036 for *P. aeruginosa*^*ATCC*^).

The median MBC (as a dilution rate from the solution containing ozone at a concentration of 0.4 mg/L) for MRSA*-Kyoto* for 1 min was 1/4 (range 1/2 to 1/4), and the median MBC for MRSA*-Kyoto* for 10 min was 1/24 (range 1/4 to 1/32). The median MBC (as an effective ozone concentration) for *P. anaerobius*^*GTC*^ (1.6 × 10^6^ CFU/mL) for 1 min was 1.1 mg/L without dilution. The air-UFB NSS did not have any bactericidal effect.

The median MBCs for *S. aureus*^*ATCC*^ for 1 min were 1/4 (range 1/4 to 1/4) for ozone-UFB NSS and < 1 for CHX. The median MBCs for *S. aureus*^*ATCC*^ for 10 min were 1/8 for ozone-UFB NSS and > 1/16 for CHX.

##### Bactericidal effects of ozone-UFB solutions at different albumin concentration

**Figure 2:**
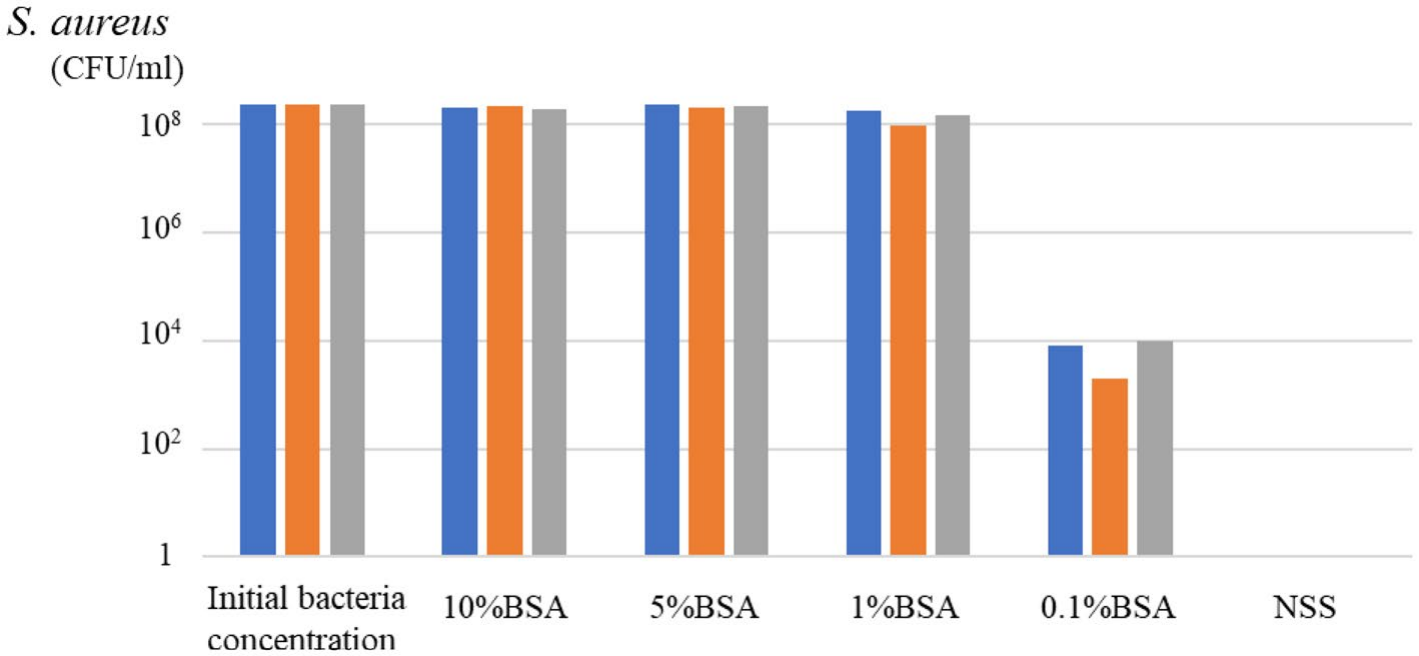
Bactericidal effects of ultrafine bubbles of ozone in a normal saline solution (NSS) on a solution of *Staphylococcus aureus* (ATCC 29213) and albumin. The ultrafine bubbles of ozone in NSS had bactericidal effects on bacteria in a solution containing albumin at a concentration of < 1 g/dL (n = 3). *BSA* bovine serum albumin.

The ozone-UFB NSS had a bactericidal effect on *S. aureus*^*ATCC*^ in a solution containing albumin at a concentration of < 1 g/dL (n = 3) (Fig. 2).

##### Bactericidal effects of ozone-UFB solutions added several times to a solution containing *S. aureus* and albumin

**Figure 3:**
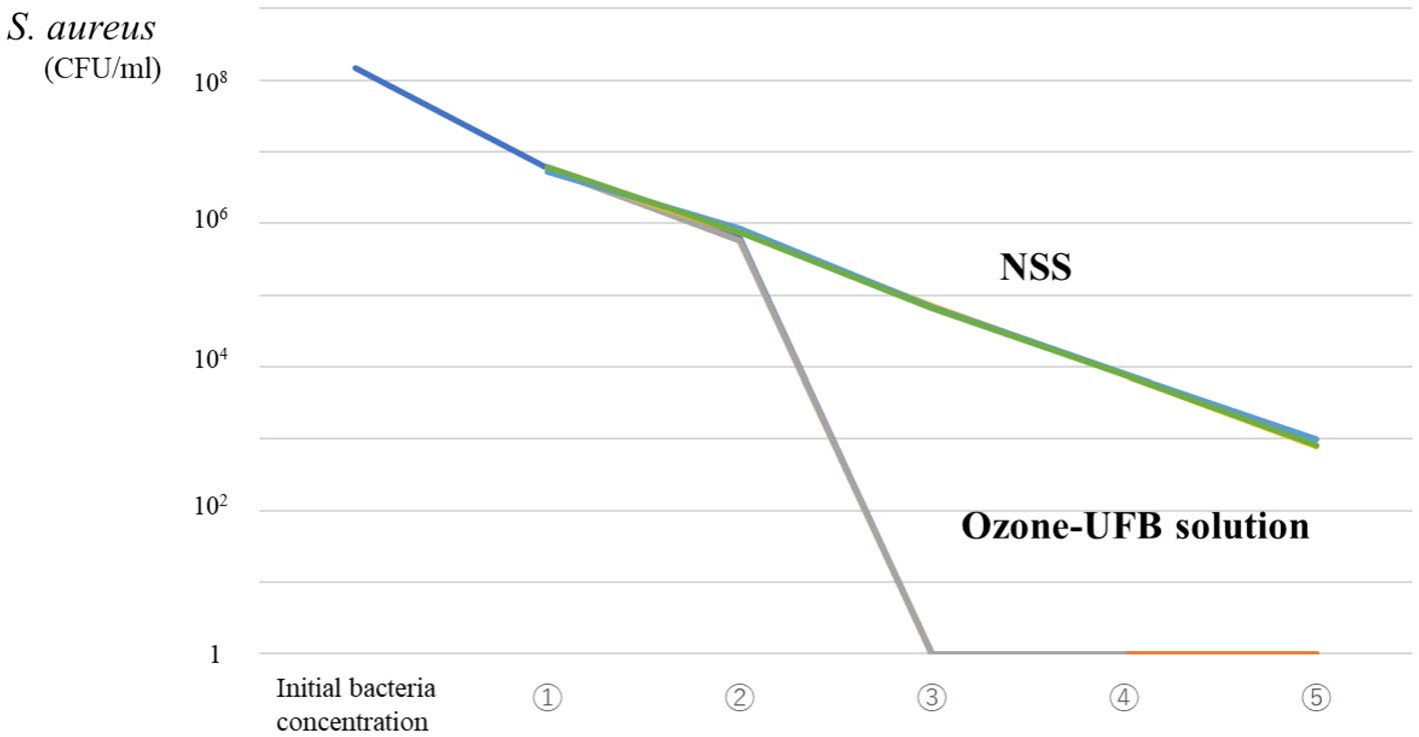
Bactericidal effects of repeated treatments with ultrafine bubbles of ozone (ozone-UFB) in normal saline solution (NSS) containing 10% albumin and *Staphylococcus aureus* (ATCC 29213). The ozone-UFB NSS had a bactericidal effect after three treatments (n = 3).

Irrigating a *S. aureus*^*ATCC*^ solution containing 10% albumin three times with ozone-UFB completely sterilized the solution, but irrigating with NSS only diluted the *S. aureus*^*ATCC*^ (n = 3) (Fig. 3).

### Rat empyema model

**Figure 4:**
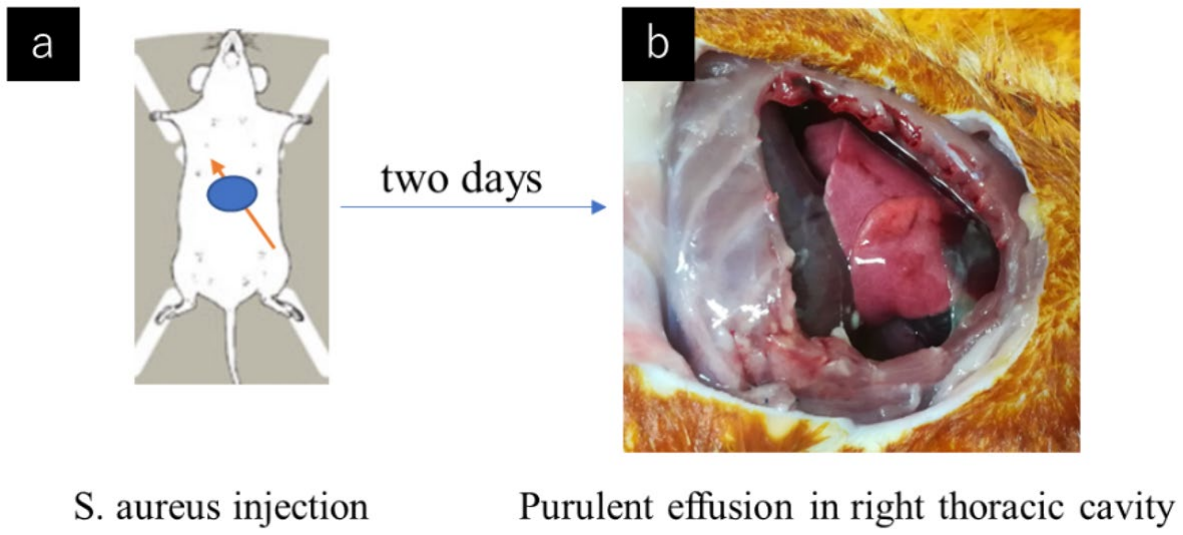
Empyema treatment in a sacrificed rat model. (**a**) *Staphylococcus aureus* (ATCC 29213) was injected into the right thoracic cavity via the diaphragm during a laparotomy. (**b**) Two days later, purulent effusion and fibrin were observed in the right and left parts of the thoracic cavity.

Solutions of *S. aureus*^*ATCC*^ (1.6 × 10^8^–3.5 × 10^8^ CFU/mL) were injected into 31 rats. Four rats died before treatment, two rats had no effusion, and eight rats had insufficient pleural effusion (< 0.5 mL). The results for 17 rats were therefore evaluated. These rats were divided into two groups, treatment with NSS (n = 10) and treatment with ozone-UFB NSS (n = 7). The appearances of the thoracic cavities 2 days after injection of *S. aureus*^*ATCC*^ are shown in Fig. 4. Purulent effusion and fibrin were observed in the right and left thoracic cavities. The volumes of pleural effusion were comparable between the NSS and ozone-UFB groups (1.3 mL [range 1.1–1.6] vs. 1.2 mL [range 1.1–1.5], *p* = 0.588). Thoracic lavage was therefore performed on both sides of the thoracic cavity through the left thoracic cavity.

**Figure 5:**
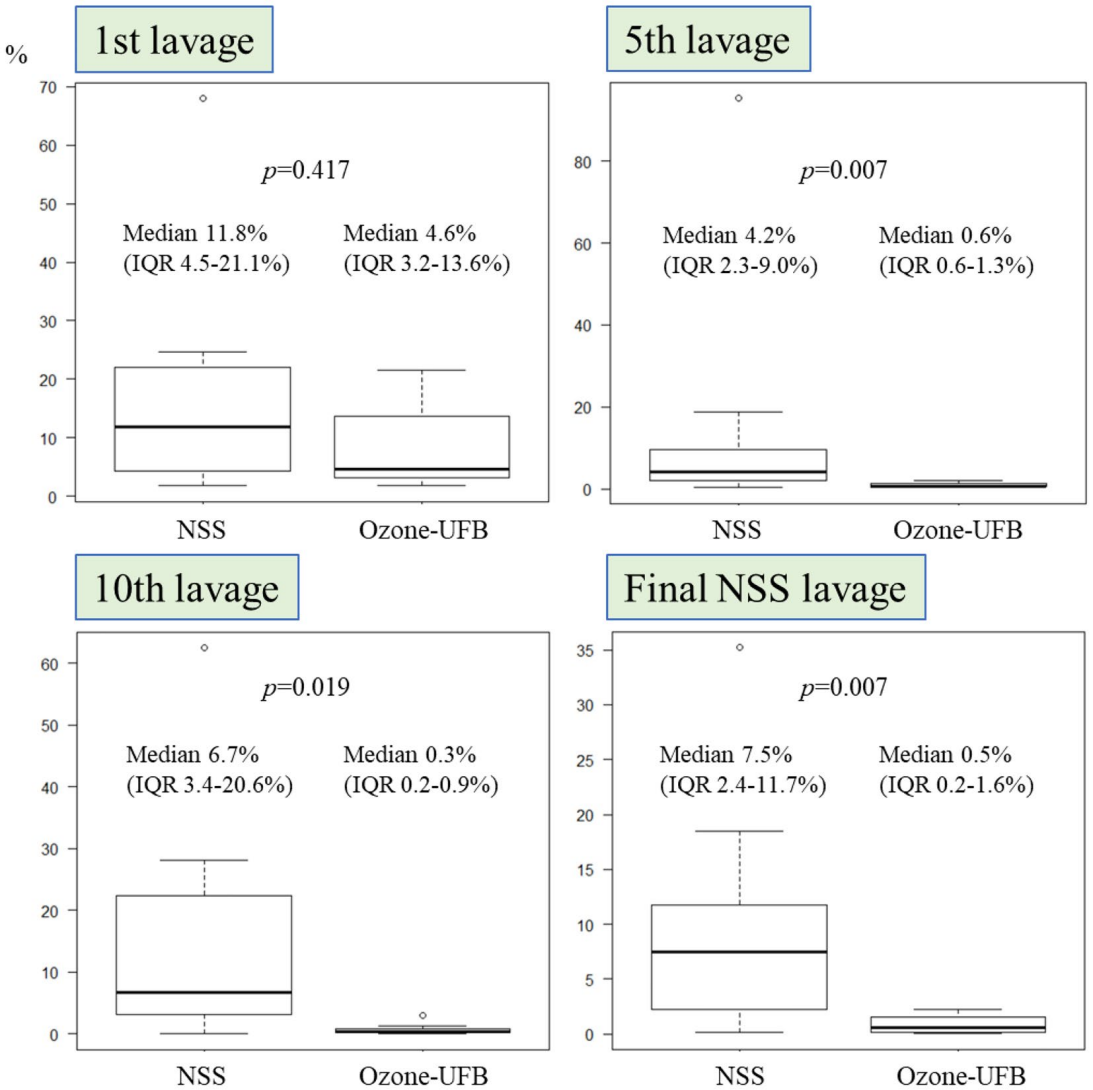
Decreases in the bacteria counts after lavage for empyema with *Staphylococcus aureus* (ATCC 29213) compared with the bacteria counts in the initial effusion. The decreases were larger in the rats treated with ultrafine bubbles of ozone (ozone-UFB) than in the rats treated with normal saline solution (NSS) after five lavages. *IQR* interquartile range.

The decrease in the bacteria count after each lavage is shown in Fig. 5. The *S. aureus*^*ATCC*^ counts for the pleural effusion and the fluid collected after urokinase lavage were decreased by 11.8% (range 4.5%–21.1%) and 4.6% (range 3.2%–13.6%), respectively, (*p* = 0.417) by the first lavage, 4.2% (range 2.3%–9.0%) and 0.6% (range 0.6%–1.3%), respectively, (*p* = 0.007) by the fifth lavage, 6.7% (range 3.4%–20.6%) and 0.3% (range 0.2%–0.9%), respectively, (*p* = 0.019) by the 10th lavage, and 7.5% (range 2.4%–11.7%) and 0.5% (range 0.2%–1.6%), respectively, (*p* = 0.007) by the last NSS lavage.

### In vivo toxicity study

**Figure 6:**
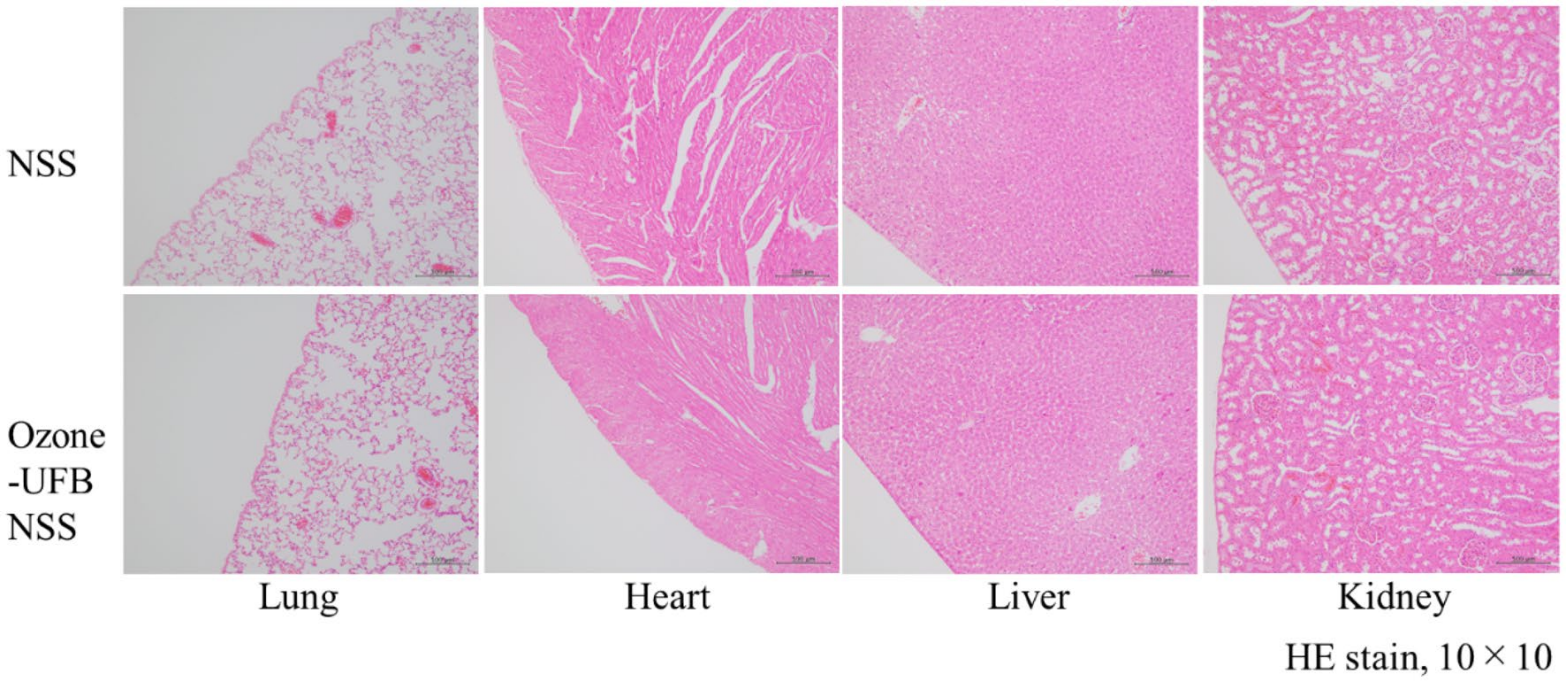
Pathological examinations of rat organs 24 h after injection of ultrafine bubbles of ozone (ozone-UFB) in normal saline solution (NSS) or NSS into the right part of the thoracic cavity (hematoxylin eosin stain, 10 × 10). There were no significant inflammatory changes in the right lower lung, heart, liver, or kidney in either the rats treated with ozone-UFBs or NSS.

Of the 16 rats that were used in the toxicity study, three were excluded because of technical damage. The 13 remaining rats were divided into two groups, NSS-treated rats (n = 6) and rats treated with ozone-UFBs (9–10 mg/L) (n = 7). All of these rats survived the evaluation period. The weights 24 h after treatment relative to the weights before treatment were 99.0% (range 98.2%–99.1%) for the NSS group and 96.0% (range 91.1%–98.1%) for the ozone-UFB group (*p* = 0.138). The serum IL-6 concentrations for all of the rats were < 5.3 pg/mL. The pathological examinations indicated that proliferation of type II alveolar epithelial cells around the airway occurred in one rat in the NSS group, focal proliferation of type II alveolar epithelial cells without infiltration of any inflammatory cells occurred in one rat in the ozone-UFB group, and aspiration pneumonia occurred in one rat in the ozone-UFB group. No inflammatory changes (e.g., pleuritis) or chemical pneumonitis, hepatitis, or nephritis was detected in any rat (Fig. 6).

## Discussion

Ozone was discovered by Christian Friedrich Schonbein in 1840, and its use in medicine was reported in the early 1900s. Ozone is a powerful oxidizing substance that has both bactericidal and cytotoxic effects. The bioavailability of ozone is considered useful as long as its capacity is not exceeded^18^.

Ozone has high sterilization ability and low persistence, and therefore, it is also used for sterilization in wastewater and food materials^12,19^. Ozone has antimicrobial effects against various bacteria and fungi^10,11^ as well as viruses such as coronavirus^20^. In the medical field, ozonated water has been applied in dental treatment^15,16^. However, because the protective ability against ozone in the respiratory epithelium is low, inhalation of ozone gas at 0.1 ppm or more must be avoided when handling ozone^18^. A method of dissolving ozone in a liquid rather than in a gaseous state has been investigated, but this strategy is limited because ozone has a short half-life and becomes unstable in liquids^10,13,14^. We developed an ozone-UFB solution with a long half-life and found that it had an excellent bactericidal effect and was safe in both in vitro and in vivo tests. The effect of ozone (non-UFB) solutions decreases immediately because the half-life is short, and readily deactivated in an organic-matter-rich environment. When using an ozone solution (non-UFB) for sterilization purposes, there has been no choice but to use it at a high concentration of diluted ozone, so far. Therefore, ozone solutions cannot be safely used as antibacterial treatments in the thoracic cavity because of the possibility of the ozone being harmful. Although lavage through a drainage tube using ozonated water at a high ozone concentration (20 mg/L) and urokinase in humans has only been performed in one previous study^14^, we found that a sufficient bactericidal effect was achieved in a sacrificed rat model by repeated lavage using the ozone-UFB solution (3–4 mg/L) even when the ozone concentration was lower than the ozone concentration in a non-UFB ozone solution used in a previous study^14^.

In addition to existing treatments such as antibiotic therapy and drainage, intrathoracic lavage can be useful to promote clearance in the thoracic cavity. We observed that performing intrathoracic lavage more than five times did not result in a proportional decrease in the rate of reduction of bacteria. To further facilitate the bactericidal effect of ozone-UFB solution, it would be possible to lower the concentration of organic matter using fibrinolytic therapies such as urokinase. We are also considering other ways to improve the bactericidal effect of ozone-UFB such as intrathoracic lavage using ozone of a higher concentration but within the safety range, and intraoperative or postoperative use with surgical treatment.

We assessed the safety of injecting the ozone-UFB solution into the chest cavities of rats, and ozone at a concentration of 9–10 mg/L was acceptable because of the pathological exam results and the IL-6 concentrations. In a previous in vitro model study in the dental field^16^ it was found that cell viability in oral tissue exposed to ozone nanobubbles in water slightly decreased over 24 h. In contrast, cell viability in oral tissue exposed to 0.2% CHX halved by 8.4 h, suggesting that CHX can damage human oral tissues and ozone nanobubbles in water would be a safer oral antiseptic.

The evaluation of safety with the application of ozone-UFBs in therapy in humans is essential. There have been several reports regarding ozone nanobubble water therapy for periodontitis^15,16^, and the low toxicity of ozone nanobubble water in gingival tissue was reported^16^. In addition, Shichiri-Negoro et al. reported that ozonated water with a diluted ozone concentration of 11 ppm could suppress *Candida albicans* growth and biofilm formation on polymethyl methacrylate without impairing surface properties^21^. A systematic review on the effect of ozone therapy in root canal disinfection reported that the antimicrobial effect depended on the concentration of ozone, treatment period, and target microorganisms^22^. Further evaluation in terms of safe and effective ozone concentrations and conditions is needed until the ozone UFBs we created using our device can be applied in treatment.

There were some limitations to the study. First, empyema is usually treated using antibiotics and drainage with or without lavage. In this study, antibiotics were not used because the aim was to evaluate the bactericidal effects of local treatment using ozone-UFBs. Second, the bactericidal effect of intrathoracic lavage using ozone-UFBs in the empyema rat model were only evaluated for *S. aureus*, which is the most common cause of empyema. We evaluated the bactericidal effects of ozone-UFBs for several types of bacteria in vitro, but further evaluations for other bacteria (e.g., other kinds of anaerobic bacteria or multi-drug resistant bacteria) will be required. Third, we did not find concentration-dependent bactericidal effects in the rat empyema model. However, it may be necessary to determine the optimal ozone concentration by investigating the relationship between the ozone concentration and bactericidal effect. Finally, the bactericidal effects were evaluated only in sacrificed rats in this study. The extent to which intrathoracic lavage using ozone-UFB solution clinically promotes empyema healing has not been verified in a living animal model. We hope to develop a long-term empyema model combined with systemic treatment and evaluate the additional effect of intrathoracic lavage using ozone-UFB solution.

## Conclusions

We found that ozone-UFBs in solution were bactericidal both in vitro and in a rat model. We also found that the effective ozone concentration was safe. This is the first paper to verify the efficacy and safety of ozone-UFBs in the body cavity.

Further studies using a living animal model to determine the therapeutic and adverse effects of ozone-UFB lavage are required before ozone-UFBs in solution can be applied to empyema in humans.

### Supplementary Information

Supplementary Figures.

## Data Availability

The datasets used and/or analysed during the current study available from the corresponding author on reasonable request.
