# Supplementary material for: Novel intrathoracic irrigation using ultrafine ozone bubbles in a rat empyema model
Source: Sci Rep. 2023 Oct 10;13:17078. doi: 10.1038/s41598-023-43787-3 (PMC10564710; doi:10.1038/s41598-023-43787-3)
Supplement: Supplementary file 1 — Supplementary Figures. [file 41598_2023_43787_MOESM1_ESM.pdf]

Supplemental Figure 1.

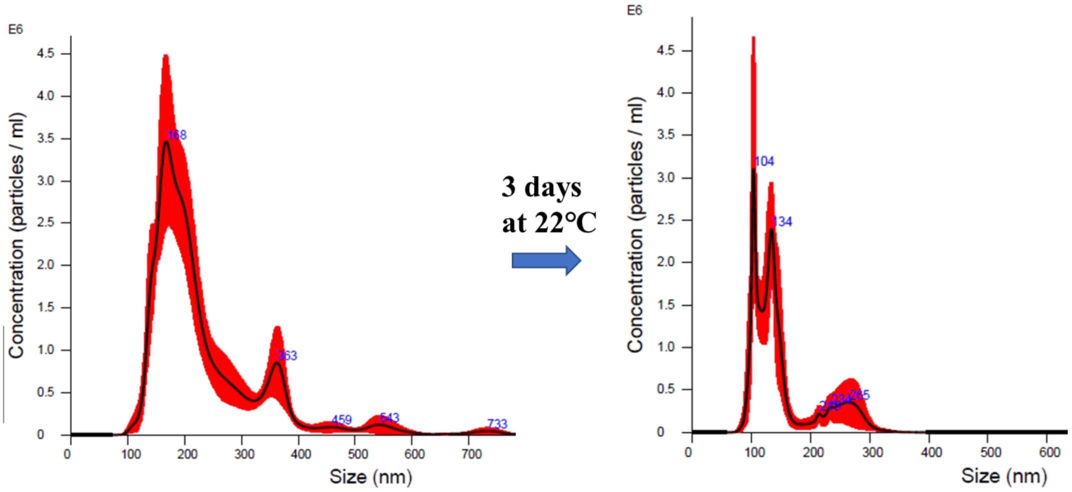

One day after production
Count of particles: 3.75×10^8/ml
Mean diameter of particles：232 nm

Four days after production
Count of particles: 1.39×10^8/ml
Mean diameter of particles: 150 nm

The number of ultrafine bubbles (UFBs) was counted using a NanoSight system (Malvern Panalytical, Malvern, UK). The number of UFBs with diameters of 100–200 nm was sufficient even 4 d after the ozone-UFBs were created in NSS at room temperature (22 °C), but larger bubbles disappeared.

Supplemental Figure 2.

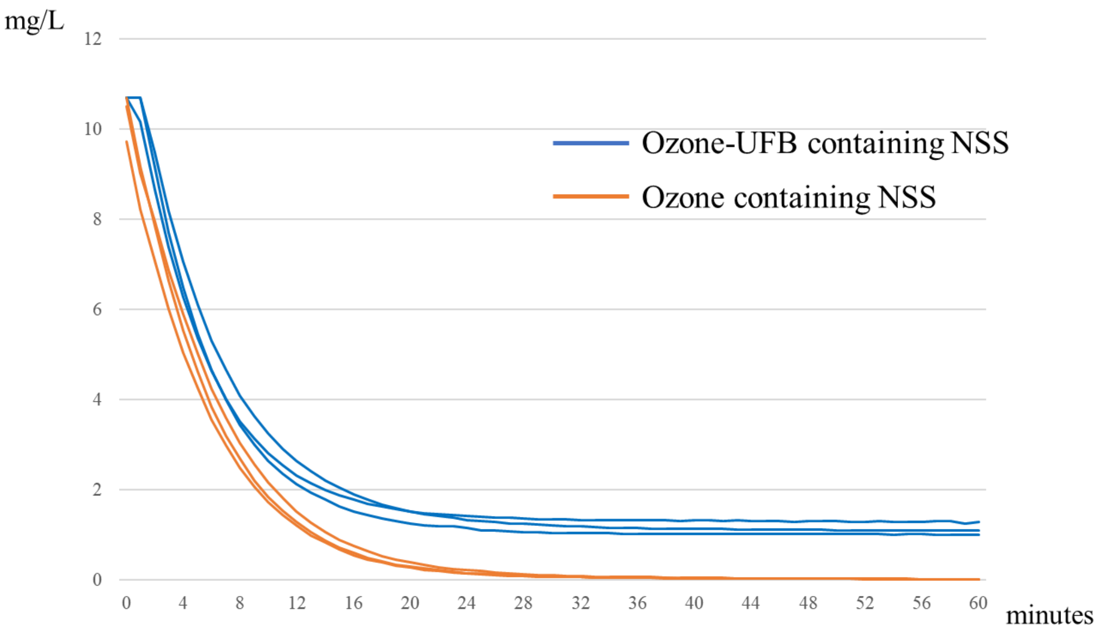

The dissolved ozone concentrations determined three times at 22 °C for 60 min after the production of each solution. The ozone concentration in the NSS containing ozone-UFBs decreased more gradually than the ozone concentration in the non-UFB ozone NSS at room temperature.
